# Concurrent Targeting of KRAS and AKT by MiR-4689 Is a Novel Treatment Against Mutant *KRAS* Colorectal Cancer

**DOI:** 10.1038/mtna.2015.5

**Published:** 2015-03-10

**Authors:** Masayuki Hiraki, Junichi Nishimura, Hidekazu Takahashi, Xin Wu, Yusuke Takahashi, Masaaki Miyo, Naohiro Nishida, Mamoru Uemura, Taishi Hata, Ichiro Takemasa, Tsunekazu Mizushima, Jae-Won Soh, Yuichiro Doki, Masaki Mori, Hirofumi Yamamoto

**Affiliations:** 1 Department of Surgery, Department of Gastroenterological Surgery, Graduate School of Medicine, Osaka University, Osaka, Japan; 2 Department of Chemistry, Biomedical Research Centre for Signal Transduction Networks, Inha University, Incheon, Korea

**Keywords:** AKT, colorectal cancer, KRAS, microRNA, miR-4689

## Abstract

*KRAS* mutations are a major cause of drug resistance to molecular-targeted therapies. Aberrant epidermal growth factor receptor (EGFR) signaling may cause dysregulation of microRNA (miRNA) and gene regulatory networks, which leads to cancer initiation and progression. To address the functional relevance of miRNAs in mutant *KRAS* cancers, we transfected exogenous *KRAS*^G12V^ into human embryonic kidney 293 and MRC5 cells with wild-type *KRAS* and *BRAF* genes, and we comprehensively profiled the dysregulated miRNAs. The result showed that mature miRNA oligonucleotide (miR)-4689, one of the significantly down-regulated miRNAs in KRAS^G12V^ overexpressed cells, was found to exhibit a potent growth-inhibitory and proapoptotic effect both *in vitro* and *in vivo*. miR-4689 expression was significantly down-regulated in cancer tissues compared to normal mucosa, and it was particularly decreased in mutant *KRAS* CRC tissues. miR-4689 directly targets v-ki-ras2 kirsten rat sarcoma viral oncogene homolog (KRAS) and v-akt murine thymoma viral oncogene homolog 1(AKT1), key components of two major branches in EGFR pathway, suggesting KRAS overdrives this signaling pathway through inhibition of miR-4689. Overall, this study provided additional evidence that mutant *KRAS* functions as a broad regulator of the EGFR signaling cascade by inhibiting miR-4689, which negatively regulates both RAS/mitogen-activated protein kinase (MAPK) and phosphoinositide 3-kinase (PI3K)/AKT pathways. These activities indicated that miR-4689 may be a promising therapeutic agent in mutant *KRAS* CRC.

## Introduction

Recent advances in our comprehension of the specific signaling pathways in cancer cells have offered novel targeted therapies for patients with metastatic colorectal cancer (mCRC). Studies have shown that monoclonal antibodies that target epidermal growth factor receptors (EGFRs) provide a survival benefit in mCRCs that harbor wild-type *KRAS*.^1,2^ A retrospective analysis of randomized, phase 2 OPUS and phase 3 CRYSTAL clinical trials revealed that cetuximab combined with a first-line chemotherapy (FOLFOX4 or FOLFIRI) significantly improved the prognosis of patients with wild-type *KRAS* mCRC compared to chemotherapy alone. However, these effects were not observed in patients with mutant *KRAS* mCRC.^2,3^ Because *KRAS* mutations occur in ~40% of patients with mCRC,^4^ novel therapeutic strategies are urgently needed for treating this population.

Ligand binding to the extracellular domain of EGFR results in phosphorylation of the tyrosine kinase located on an intracellular domain. This activation of the receptor then induces the activation of intracellular effectors via intracellular signaling pathways, including one where rapidly accelerated fibrosarcoma (RAF) activates the dual kinase, mitogen-activated protein kinase (MAPK)/extracellular signal-regulated kinase (ERK) kinase (MEK), which then activates the ERK pathway. In addition, EGFR signals via the stress-activated protein kinase (SAPK)/c-Jun N-terminal kinase (JNK) pathway, the phosphoinositide 3-kinase (PI3K)/AKT pathway, the signal transducers and activators of transcription (STAT) 3 pathway, and the phospholipase Cγ pathway.^5,6,7^ Cetuximab binds to EGFR with a high specificity and blocks ligand-induced phosphorylation of the receptor. This blocking leads to the inhibition of EGFR downstream signaling pathways, including the RAF/MEK/ERK pathway, which is mainly involved in cell proliferation, and the PI3K/AKT pathway, which is mainly involved in cell survival and tumor invasion.^8,9^ However, once point mutations in the *KRAS* oncogene occur, the downstream pathways become constitutively active (RAF/MEK/ERK, SAPK/JNK, and possibly PI3K/AKT pathways).^10^ This constitutive activity is considered the major reason that mutant *KRAS* CRCs are resistant to anti-EGFR therapy.^1,7^

Combination therapies with multiple inhibitors are currently in development to overcome resistance to anti-EGFR therapy. However, a clinical trial for testing the MEK1/2 inhibitor, PD0325901,was discontinued in phase 2 because of severe toxicity, which included blurred vision and acute neurotoxicity.^11,12^ Another MEK1/2 inhibitor, RDEA119, combined with sorafenib also led to severe adverse events, including liver failure, sepsis/hepatic encephalopathy, and tumor lysis syndrome for patients with hepatocellular carcinoma in a phase 2 study.^13^ A multicenter phase 1/2 study to evaluate another MEK1/2 inhibitor, AS703026, plus 5-fluorouracil, leucovorin, and irinotecan (FOLFIRI) for mutated *KRAS* mCRC was unable to proceed to phase 2, because effective doses of AS703026 could not be achieved due to its toxicity.^14^ Therefore, novel effective therapeutic alternatives are critically needed.

MicroRNAs (miRNAs) are endogenous, single-stranded, small, noncoding RNAs of 20–22 nucleotides. miRNAs bind to complementary sequences, mainly in the 3′-untranslated regions (3′-UTR), of multiple target mRNAs, and they can either cause translational repression or facilitate cleavage of their target mRNAs.^15,16^ miRNAs are involved in crucial biological processes, including development, differentiation, apoptosis, and proliferation.^17,18^ To date, a few studies have shown, through searches with Target Scan and/or sequence binding prediction programs, that miR-30b, miR-18a, and miR-143 specifically inhibit KRAS expression in colon cancer cells.^19,20,21^

This study aimed to discover a powerful, therapeutic miRNA that could target mutant *KRAS* in CRC. For this purpose, we performed a microarray analysis in *KRAS*-transfected, human embryonic kidney 293 (HEK293) and human lung fibroblasts (MRC5). We screened the ability of candidate miRNAs to suppress signal transduction in the RAF/MEK/ERK and MEKK/SEK/JNK pathways. Because miRNAs can bind to multiple target regions through imperfect complementarity,^22^ we also investigated whether candidate miRNAs could concurrently inhibit other transduction pathways, such as the PI3K/AKT and STAT3 pathways.

## Results

### KRAS^G12V^ overexpression downregulates a subset of miRNAs

**Figure 1:**
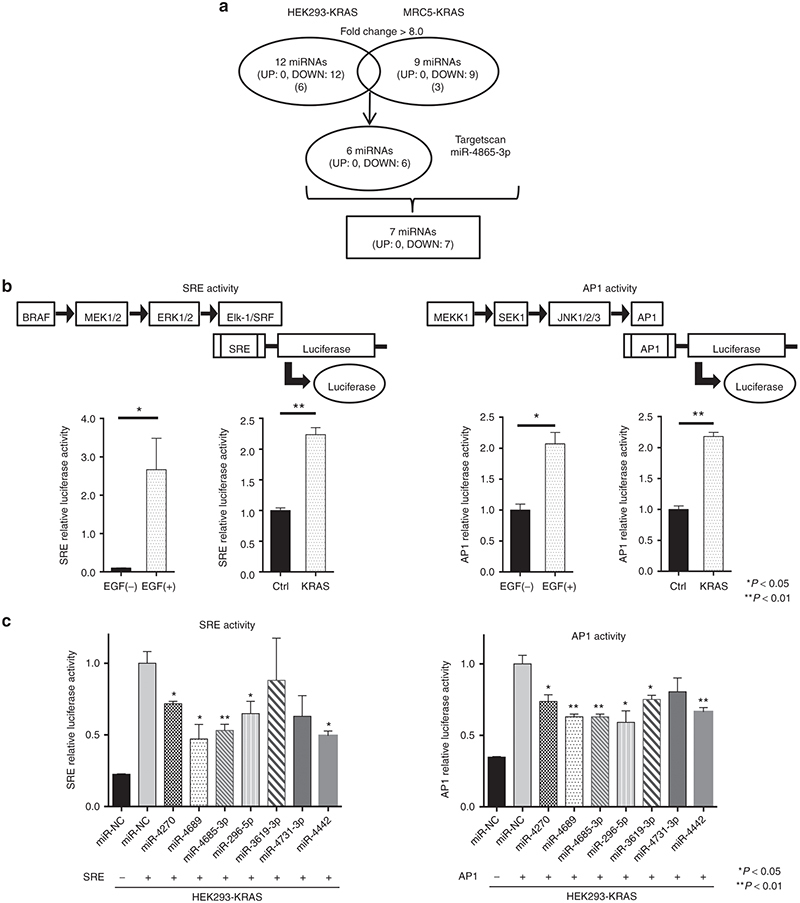
**Screening for candidate miRs that could suppress SRE or AP1 transcription activity in HEK293-KRAS.** Mutant *KRAS* cDNA was introduced into HEK293 or MRC5 cells. MiRNA expression profiles were compared between these cells with a miRNA microarray analysis (see **Supplementary Figure S1**). (**a**) Schematic diagram of the results of microarray analysis. The protocol identified 6 miRNAs common to both cell lines that showed >8.0-fold decreased expression in HEK293-KRAS and MRC5-KRAS compared to control HEK293 and MRC5 cells. In addition to those six miRNAs, we identified miR-4685-3p with a Target Scan search, which indicated that it was likely to bind to the 3′UTR region of MEK2, and it functioned downstream of the KRAS signaling pathway (see **Supplementary Table S1 and S2**). (**b**) Activated KRAS signaling through (left) RAF-MEK-ERK and (right) MEKK1-SEK1-JNK transduction pathways. Addition of EGF or introduction of a mutant *KRAS* gene into HEK293 enhanced (left) SRE and (right) AP1 transcription of the luciferase reporter gene. (**c**) Screening candidate miRs to identify those that inhibit the two signal transduction pathways. Several miRNAs significantly suppressed SRE or AP1 transcription of luciferase compared with miR-NC in HEK293-KRAS cells. We selected miR-4689, miR4685-3p, miR-296-5p, and miR-4442 for further evaluations in mutant *KRAS* CRC cells. All data represent the mean ± SEM; * *P* < 0.05, ** *P* < 0.01.

We first examined whether mutated *KRAS* could induce dysregulation of miRNA expression. With a miRNA microarray, we compared the profile of miRNAs expressed in HEK293 and MRC5 cells that overexpressed *KRAS*^G12V^ to those expressed in parental cells that harbored wild-type *KRAS*. The results indicated that a subset of miRNAs, including miR-4270, miR-4689, miR-296-5p, miR-3619-3p, miR-4731-3p, and miR-4442, was significantly down-regulated in *KRAS*^G12V^ transfected cells. Most of these miRNAs were commonly down-regulated in both cell types (see **Supplementary Figure S1 and Supplementary Tables S1 and S2**). We also included miR-4685-3p for comparison in further analyses, because miR-4685-3p potentially targeted MEK2, a downstream target of KRAS, based on an *in silico* prediction obtained with Target Scan 6.2 software^23^ (**Figure 1a**).

### miR-4689 inhibits KRAS signaling activity

**Figure 2:**
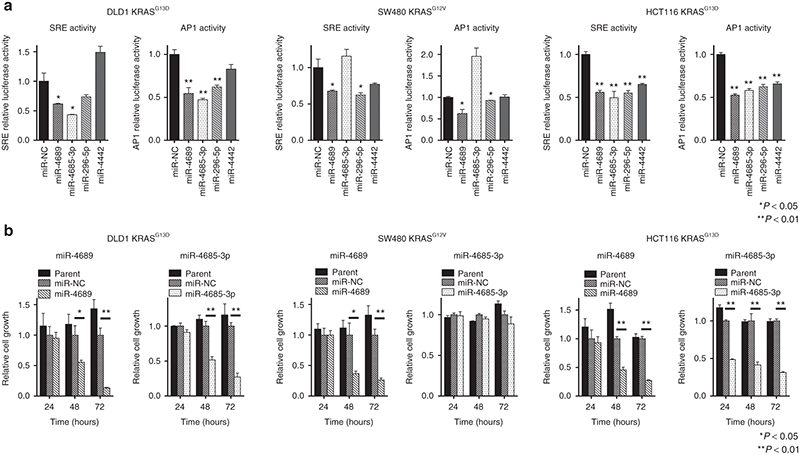
**Inhibitory effects of miRs on SRE or AP1 *in vitro* transcription activity and on proliferation of mutant *KRAS* CRC cell lines.** (**a**) Effects of four miRs on SRE or AP1 transcription of luciferase in mutant *KRAS* cell lines. (**b**) Effects of miR-4689 and miR-4685-3p on proliferation of mutant *KRAS* CRC cells. All data represent the mean ± SEM. *P < 0.05, **P < 0.01.

To determine the functional relevance of these miRNAs in KRAS signaling, we used serum response element (SRE) and activator protein-1 (AP1) reporter assays. The pGL4.33[luc2P/SRE/Hygro] vector contained SRE, which drives the transcription of the luciferase reporter gene, *luc2P*, in response to activation of the MAPK/ERK signaling pathway.^24^ The pGL4.44[luc2P/AP1 RE/Hygro] vector contained an AP1 response element that drives the transcription of *luc2P*, in response to activation of the MAPK/JNK signaling pathway. We confirmed that the addition of EGF or the introduction of a mutant *KRAS* significantly enhanced both SRE and AP1 transcription activities in HEK293 cells (**Figure 1b**). In HEK293 cells that overexpressed *KRAS*^ G12V^ (HEK293-KRAS), most of the tested miRNAs inhibited SRE and AP1 transcriptional activities, except miR-3619-3p (in SRE-reporter cells) and miR-4731-3p (in AP1-reporter cells) (**Figure 1c**). Further analyses in mutant *KRAS* CRC cell lines, including the SW480 (KRAS^G12V^), DLD1 (KRAS^G13D^), and HCT116 (KRAS^G13D^) lines, revealed that only miR-4689 potently suppressed both SRE and AP1 transcriptional activities in all tested mutated *KRAS* cell lines (**Figure 2a**). Furthermore, miR-4689, but not miR-4685-3p, significantly inhibited cell proliferation in these cell lines (**Figure 2b**).

### miR-4689 is downregulated in mutant *KRAS* CRC

**Figure 3:**
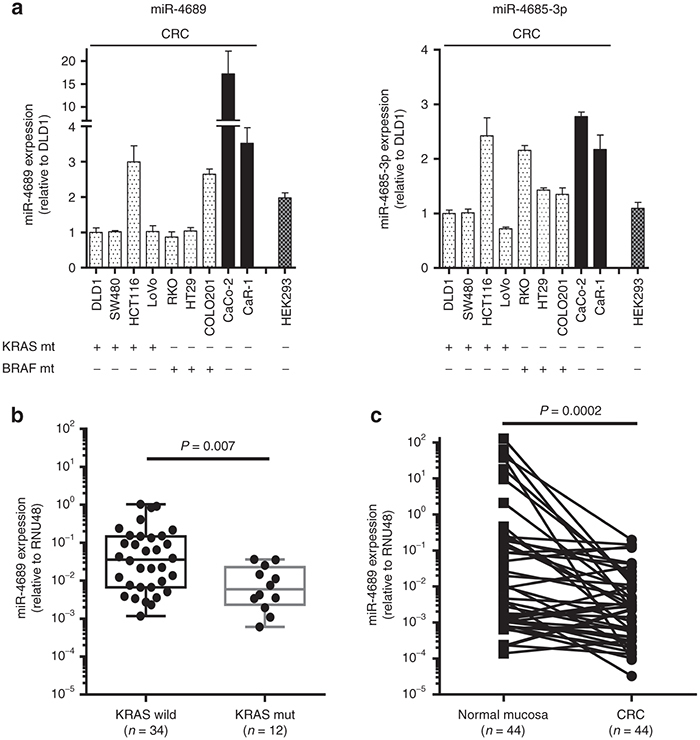
**Expression of miRs in cell lines and clinical tissue samples.** (**a**) Expression of miR-4689 and miR-4685-3p in mutated *KRAS* or *BRAF* CRC cell lines (cell genotypes indicated below the graphs). (Left) miR-4689 expression was lower in cell lines with mutated *KRAS* or *BRAF* (speckled bars) than in cells with wild-type *KRAS* or *BRAF* (filled bars). (Right) miR-4685-3p expression was similar in cell lines with mutated *KRAS* or *BRAF* (speckled bars) and cells with wild-type *KRAS* or *BRAF* (filled bars). Results represent the mean ± SEM. (**b**) Expression of miR-4689 in mutant (mut; *n* = 12) and wild-type (wild; *n* = 34) *KRAS* CRC clinical samples. miR-4689 expression was significantly lower in mutant than in wild-type *KRAS* CRCs (*P* = 0.007). (**c**) Expression of miR-4689 was significantly higher in normal colonic mucosa than in CRC tissue samples (*P* = 0.002)

Next, we compared the expression of miR-4689 in nine CRC cell lines of which seven cell lines were mutated *KRAS* or *BRAF* cells. Wild-type *KRAS* or *BRAF* cell lines had significantly higher miR-4689 levels than did mutated *KRAS* or *BRAF* CRC cell lines (**Figure 3a**, *P* = 0.02). miR-4689 was downregulated in mutant *KRAS* CRC clinical samples when compared with wild-type *KRAS* samples (**Figure 3b**, *P* = 0.007). Consistent with its tumor suppressive properties, the expression of miR-4689 was significantly downregulated in CRC tissues compared with its expression in normal mucosa (**Figure 3c**, *P* = 0.0002).

*In vitro* mechanistic studies confirmed an inverse correlation between KRAS and miR-4689 expression levels. Thus, forced expression of *KRAS* gene in nontumor KEK293 and MRC5 significantly decreased miR4689 expression (see **Supplementary Figure S2a,b**, *P* = 0.018 and 0.03, respectively). In contrast, knockdown of *KRAS* gene expression with the shKRAS vector increased miR-4689 expression in *KRAS* mutated DLD1 cells (*P* = 0.002, see **Supplementary Figure S3a**).

### miR-4689 directly targets KRAS mRNA

**Figure 4:**
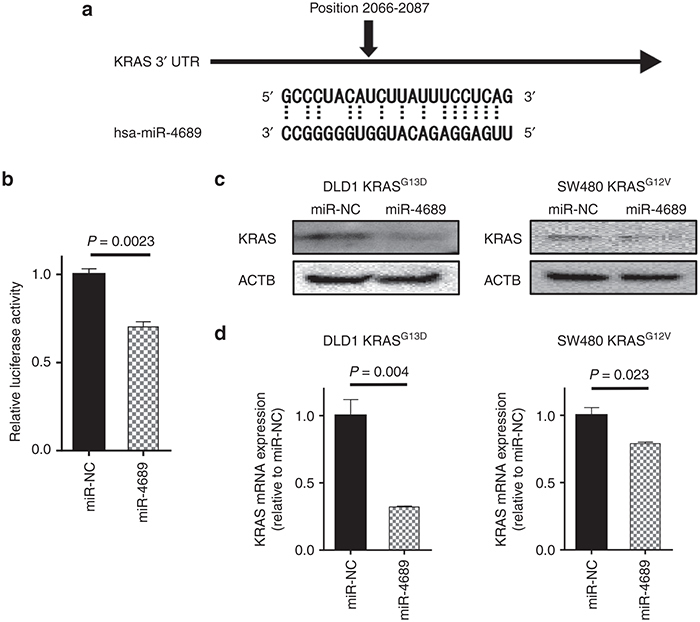
**KRAS is a direct target of miR-4689 in mutant *KRAS* CRC cells.** (**a**) Schematic of the human KRAS 3′-UTR; sequence alignment with miR-4689 shows complementary nucleotides for binding (dotted lines). (**b**) Luciferase activity of the KRAS-3′UTR reporter in the presence of 50 nM of miR-4689 or miR-NC. (**c**) Immunoblots of KRAS in SW480 KRAS^G12V^ and DLD1 KRAS^G13D^ cells treated with miR-NC or miR-4689. Actin-beta (ACTB) is the loading control. (**d**) qRT-PCR results show relative expression of KRAS mRNAs in SW480 KRAS^G12V^ and DLD1 KRAS^G13D^ cells treated with miR-NC or miR-4689. All data represent mean ± SEM.

To gain further insight into how miR-4689 regulated the KRAS signaling pathway, we searched for potential targets of miR-4689 among the signal transduction components. We found that KRAS possessed a putative binding site for miR-4689 on the 3′-UTR of its mRNA (**Figure 4a**). Moreover, miR-4689 overexpression inhibited the luciferase reporter activity of a plasmid that included the 3′-UTR region of KRAS (**Figure 4b**). Furthermore, miR-4689 inhibited KRAS expression, at both the protein (**Figure 4c**) and mRNA (**Figure 4d**) levels.

### AKT1 is also a target of miR-4689

**Figure 5:**
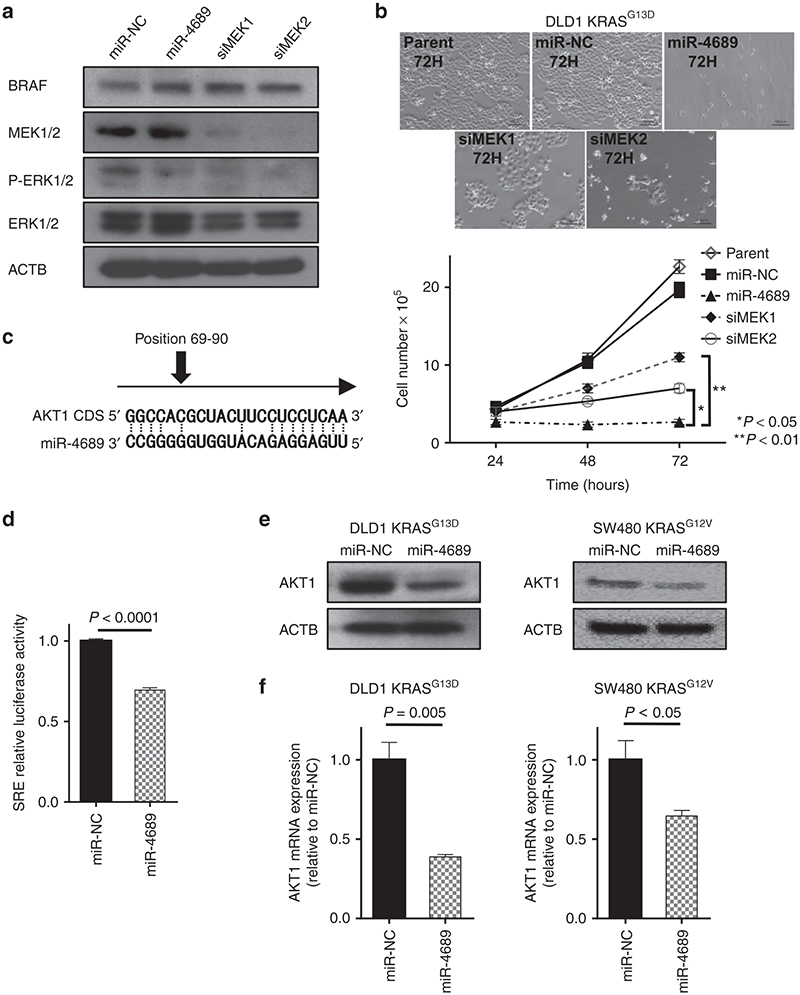
**AKT1 is a direct target of miR-4689 in mutant *KRAS* CRC cells.** (**a**) ERK phosphorylation was similar in DLD1 KRAS^G13D^ cells treated with miR-4689, siMEK1, or siMEK2 (25 nM). (**b**) Proliferation of mutated *KRAS* DLD1 cells was significantly suppressed by miR-4689 compared to siMEK1 or siMEK2 (*P* < 0.01 and *P* < 0.05, respectively, two-way ANOVA). (**c**) Sequence alignment of miR-4689 with the coding sequence (CDS) of human AKT1 shows complementary nucleotide binding sites (dotted lines). (**d**) Luciferase activity of the AKT1 CDS reporter in the presence of 50 nM of miR-4689 or miR-NC. (**e**) Immunoblots and (**f**) qRT-PCR results show AKT1 expression in SW480 KRAS^G12V^ and DLD1 KRAS^G13D^ cells treated with miR-NC or miR-4689. ACTB was the housekeeping control. All data represent mean ± SEM.

Although miR-4689 and siRNA-mediated silencing of MEK1 and MEK2 had similar effects on ERK phosphorylation (**Figure 5a**), miR-4689 inhibited proliferation more potently than the siRNA treatment in mutated *KRAS* DLD1 cells (**Figure 5b**). This raised the possibility that miR-4689 might target additional proliferation-associated genes outside of the MAPK/ERK pathway. We first searched for a miR-4689 binding site on the 3′UTRs of key molecules in other pathways related to EGFR signaling; however, no putative sequence was found. Recent evidence showed that, in addition to 3′UTRs, miRNAs can target open reading frames (ORFs) and 5′UTRs of mRNAs to regulate gene expression.^25,26,27^ Further investigation of related molecules showed that the *AKT1* gene, a crucial component of the PI3K pathway, carried a highly conserved miR-4689 binding element on its ORF sequence (nucleotides 69–90; **Figure 5c**). Luciferase activity of a reporter plasmid that harbored the *AKT1* ORF sequence was significantly inhibited with overexpression of miR-4689; this result suggested that miR-4689 might directly bind the coding sequence of AKT1 mRNA (**Figure 5d**). Inhibition of the *AKT1* gene by miR-4689 was further confirmed in Western blot (**Figure 5e**) and real-time quantitative reverse-transcription PCR (qRT-PCR) (**Figure 5f**) assays.

### miR-4689 induces apoptosis in mutant *KRAS* cells

**Figure 6:**
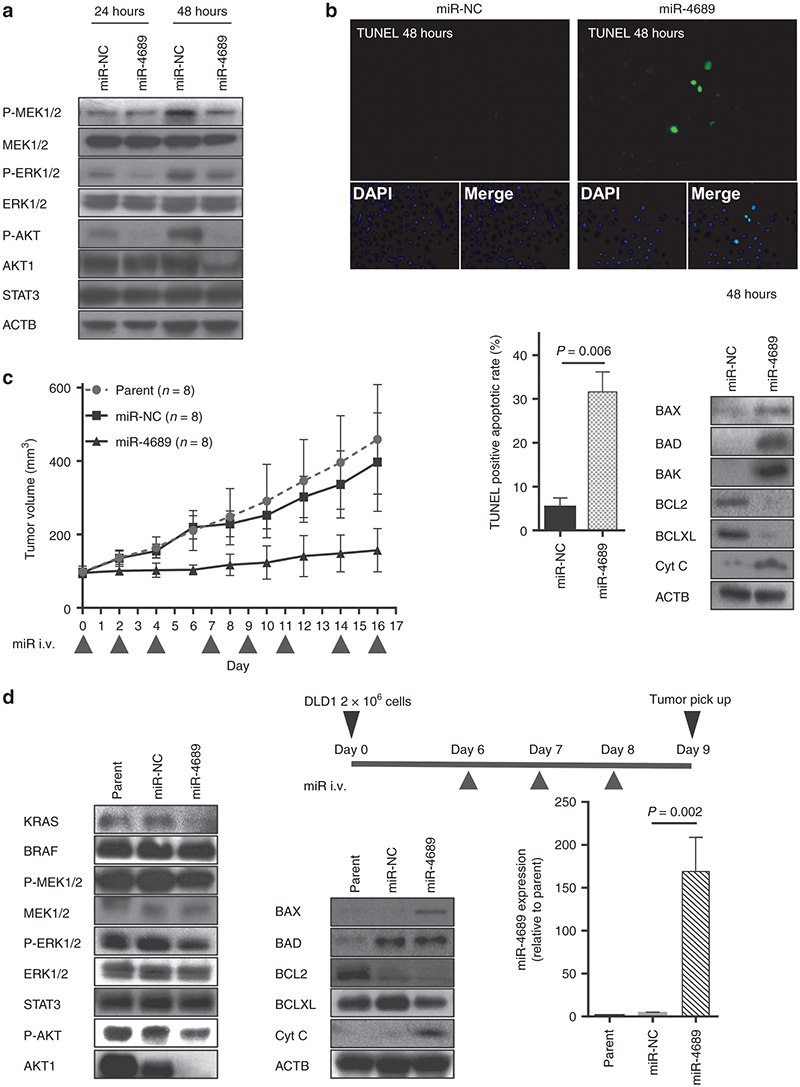
**miR-4689 affects both RAF/MEK/ERK and PI3K/AKT pathways and induces apoptosis in mutant *KRAS* CRC cells.** (**a**) Immunoblots show BRAF, P-MEK1/2, MEK1/2, P-ERK, ERK, P-AKT, AKT1, and STAT3 expression in KRAS^G13D^ DLD1 cells treated with miR-NC or miR-4689. ACTB is a loading control. Lipofectamine-mixed culture medium was exchanged with a fresh medium containing 10% FBS at 24 hours after transfection. (**b**) Apoptosis induced with miR-4689. (Top and Lower left) TUNEL staining (green) shows increased numbers of apoptotic KRAS^G13D^ DLD1 cells treated with miR-4689 compared to those treated with miR-NC. (Lower right) Immunoblots in whole cells show BAX, BAD, BAK, BCL2, BCLXL, and Cyt C expression in KRAS^G13D^ DLD1 cells treated with miR-NC (50 nM) or miR-4689 (50 nM). ACTB is a loading control. (**c**) Systemic delivery of formulated miR-4689 *in vivo* inhibited growth of established CRC tumors. Nude mice models with xenografted KRAS^G13D^ DLD1 tumors, received systemic administrations of carbonate apatite-formulated miR-4689 or miR-NC on days 0, 2, 4, 7, 9, 11, 14, and 16 via a tail vein injection (i.v., indicated below the X-axis). Each shot contained 40 µg of formulated oligo, with a 40% conjugation rate. Tumor volumes were assessed for the parent (no treated) (*n* = 8), miR-NC treated (*n* = 8), and miR-4689 treated (*n* = 8) groups on days 0, 2, 4, 6, 8, 10, 12, 14, and 16. All data represent the mean ± SEM (*P* < 0.0001, two-way ANOVA). (**d**) Characteristics of tumors induced *in vivo* by injection of KRAS^G13D^ DLD1cells. (Top) Diagram showing treatment protocol. (Day 0) Nude mice received bilateral, subcutaneous injections of KRAS^G13D^ DLD1cells (2 × 10^6^ cells) into the lower back region. Then, mice were injected via the tail vein with carbonate apatite-formulated miR-4689 or miR-NC on days 6, 7, and 8 after the cell injection. Tumors were resected on day 9. (Lower left and middle) Immunoblots show KRAS, BRAF, P-MEK1/2, MEK1/2, P-ERK1/2, ERK1/2, STAT3, P-AKT, AKT1, BAX, BAD, BCL2, BCLXL, and Cyt C expression in whole tumors excised from parent (no treated), miR-NC treated, and miR-4689 treated mice. (Lower right) Expression of miR-4689 in tumors treated with miR-4689 was significantly higher than that of tumors treated with miR-NC (*P* = 0.002). Data represent the mean ± SEM; *n* = 3 for each group.

Because miR-4689 could regulate both MAPK/ERK and PI3K/AKT pathways, we next investigated whether miR-4689 had a proapoptotic effect in mutated *KRAS* DLD1 cells. Transfection of miR-4689 reduced the levels of phosphorylated ERK and phosphorylated AKT (**Figure 6a**), and increased the number of Terminal Deoxynucleotidyl Transferase–Mediated dUTP Nick-End Labeling (TUNEL)-positive apoptotic cells (**Figure 6b**) *in vitro*. Overexpression of miR-4689 induced the expression of proapoptotic genes, *BAX*, *BAD*, *BAK*, and *Cyt C*, and suppressed the expression of antiapoptotic genes, *BCL2* and *BCLXL* (**Figure 6b**). In contrast, knockdown of KRAS or AKT1 alone led to only limited effects on altered expression of the apoptosis-related genes (see **Supplementary Figure S4**). These results imply that targeting both KRAS and AKT1 by miR-4689 is essentially important to induce apoptosis in mutated *KRAS* DLD1 cells.

### Systemic delivery of formulated miR-4689 potently inhibits tumor growth *in vivo*

Finally, we investigated the *in vivo* tumor inhibitory effect of miR-4689 with systemic administration in a mouse model, which is more feasible than focal injection for evaluating drug delivery and efficiency. We used carbonate apatite as the vehicle^28,29,30^ via tail vein injections. The systemic administration of this formulated miR-4689 dramatically inhibited tumor growth in a pre-established mouse xenograft (**Figure 6c**, *P* < 0.0001). We confirmed that the expression of KRAS, AKT1, and other crucial components of the MAPK/ERK and PI3K/AKT signaling pathways decreased in mouse xenografts (Day 9, after three single systemic administrations of miRNAs; **Figure 6d**). Xenografts, which were exposed to the systemic miRNAs, exhibited ~38.8-fold higher concentrations of miR-4689 compared to the negative control; this result indicated efficient drug delivery to the focal site (**Figure 6d**, *P* = 0.002).

### *In vivo* safety test of miR-4689

**Figure 7:**
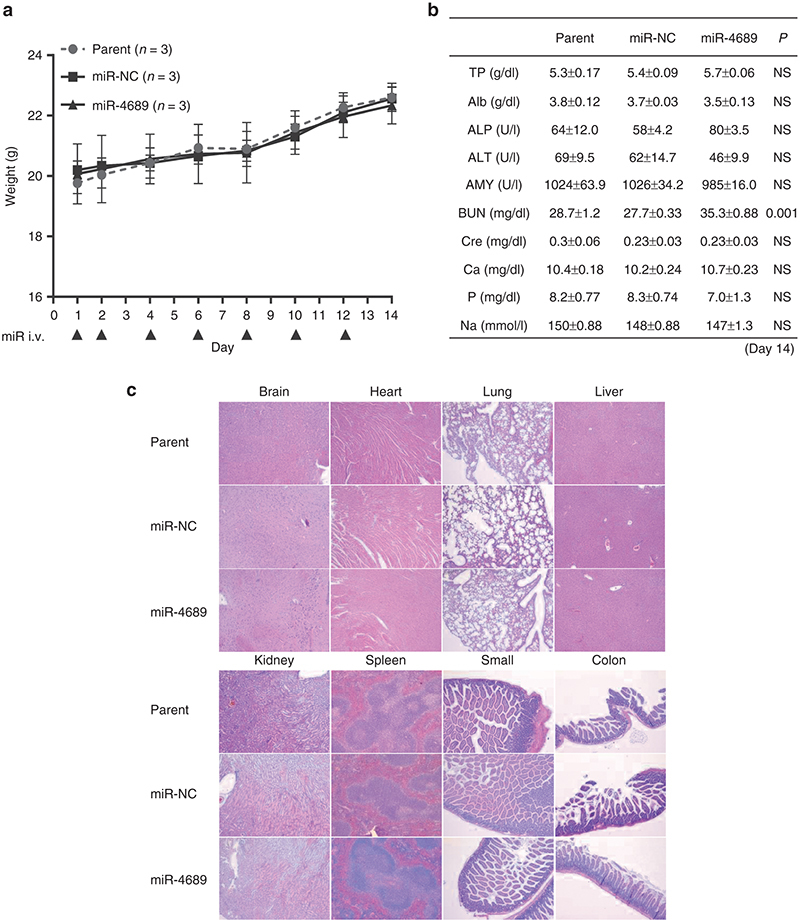
**Assessment of the toxicity of miR-4689 i*n vivo*.** Nude mice with no tumor received systemic administrations of carbonate apatite-formulated miR-4689 or miR-NC on days 1, 2, 4, 6, 8, 10, and 12 via a tail vein injection (The arrows indicates injection). Each shot contained 40 µg of formulated oligo. (**a**) Body weight loss was assessed for the parent (no treated) (*n* = 3), miR-NC treated (*n* = 3), and miR-4689 treated (*n* = 3) groups on days 1, 2, 4, 6, 8, 10, 12, and 14. (**b**) Biochemical blood examination on day 14 was performed in Parent, miR-NC and miR-4689 groups. NS = not significant (**c**) Histological findings (HE stain ×40) demonstrated that the systemic administration of miR-4689 had no serious adverse effects on each organ tissue (brain, heart, lung, liver, kidney, spleen, small intestine, and colon). Data represent the mean ± SEM; *n* = 3 for each group.

To examine *in vivo* safety, we investigated the toxicity of repeat administration of miR-4689 (40 g per injection × 7 times) at day 14. We observed no mortalities or body weight loss (**Figure 7a**). Blood chemistry tests revealed no physiologically significant differences between miRNA negative control oligonucleotide (miR-NC) and miR-4689 treated groups (**Figure 7b**), except for the slight increase in BUN (*P* = 0.001). Hematoxylin and eosin-stained sections of each organ tissue (brain, heart, lung, liver, kidney, spleen, small intestine, and colon) showed that miR-4689 caused no particular histological damage (**Figure 7c**).

## Discussion

In this study, we focused on dysregulated miRNAs in oncogenic *KRAS*-driven CRC. Earlier studies have identified miRNAs that targeted components of the EGFR signaling pathway^19,20,21^; however, few studies have explored the miRNA alterations in mutated *KRAS* tumors and their impact on cancer progression.

To identify mutant *KRAS*-related miRNAs, we introduced *KRAS*^G12V^ into noncancerous HEK293 and MRC5 cell lines, rather than wild-type *KRAS* CRC cells. This strategy allowed us to exclude numerous potential background disorders of miRNAs, caused by various genetic and epigenetic alterations observed in cancer, which facilitated the identification of miRNAs induced solely by *KRAS* gene alterations.

We selected for therapeutic miRNAs, mainly by evaluating the RAF/MEK/ERK pathway, and measuring SRE activity after treatment with specific miRNAs. Inhibition of the MAPK/ERK pathway is a minimum requirement for suppression of mutant *KRAS* tumor cells. Indeed, Migliardi *et al.*^31^ showed that cotreatment with a MEK inhibitor and a PI3K/mTOR inhibitor suppressed tumor growth in patient-derived xenografts of mutant *KRAS* colorectal carcinomas. Misale *et al.*^32^ showed that the cetuximab-resistant DiFi and Lim1215 cells recovered sensitivity in response to combinatorial targeting of MEK and EGFR. Lee *et al.*^33^ reported that combination treatment with simvastatin, which inhibits the MAPK/ERK pathway, and cetuximab showed synergistic antitumor effects in xenograft tumors that originated from mutant *KRAS* CRC cells. Those findings suggested that combined blockade of multiple pathways is critical for targeting tumors that harbor *KRAS* mutations.

After screening for SRE/AP1 activity, we identified miR-4689 and miR-4685-3p as potent inhibitors in the RAF/MEK/ERK or MEKK/SEK/JNK pathway. It is of interest that miR-4689, but not miR-4685-3p, inhibited proliferation of mutant *KRAS* SW480 cells, and that only miR-4689 could correct altered RAF/MEK/ERK activity (**Figure 2**). Based on these findings, an investigation is currently underway to pursue the possibility of developing a tailor-made medicine based on reporter assays performed with surgically resected tumor cells. The *in vitro* proliferation assay showed that miR-4689 potently inhibited cell growth in all tested CRC cell lines with *KRAS* mutations. To our knowledge, this was the first report to describe miR-4689 function in cancer development.

Our results showed that miR-4689 could directly bind to the 3′UTR region of KRAS and to the ORF of AKT1 mRNAs. It is known that miRNAs preferably bind to 3′UTR regions to regulate gene expression; however, recent evidence had suggested that ORFs and 5′ UTRs also included numerous miRNA binding elements, and the binding of miRNAs to these regions could lead to activation or inhibition of target mRNAs.^25,26,27^ A few well-understood examples include miR143, which binds to the ORF of *BRAF* mRNA, and miR145, which binds to the ORF of DNA Fragmentation Factor-45 (*DFF45*) mRNA, to regulate gene expression.^20,34^ In our experiments, miR-4689 inhibited the luminescence intensity of a luciferase reporter that carried a miR-4689 binding element from the *AKT1* coding region; this result indicated that miR-4689 directly interacted with the *AKT1* ORF (**Figure 5d**). We found that miR-4689 could significantly inhibit AKT1 expression, at both the mRNA and protein levels (**Figure 5e**,**f**).

Earlier studies have shown that targeting either the RAF/MEK/ERK or the PI3K/AKT pathway alone was not sufficient to regulate the entire EGFR signaling pathway, because one signal transduction branch could compensate for diminished signaling in another branch. For example, a selective MEK inhibitor was shown to induce activation of AKT in basal-like breast cancer models and in pancreatic cancer cell lines.^35,36,37^ In addition, PI3K/AKT pathway activation caused resistance to MEK inhibitors in mutant *KRAS* cells.^38^ Indeed, we validated that knockdown of KRAS alone could not down-regulate the ERK phosphorylation in DLD1 and that it led to only limited growth inhibition, which is consistent with an earlier finding reported by Singh *et al*.^39^ (see **Supplementary Figure S3b**). In this regard, the ability of miR-4689 to target both RAF/MEK/ERK and PI3K/AKT pathways through direct inhibition of KRAS and AKT1 represents a promising strategy against mutant *KRAS* tumors. We further demonstrated that cotransfection of miR-4689 together with a KRAS- and/or AKT-expression vector in DLD1 restored cell proliferation (see **Supplementary Figure S5**). This result indicates that the targeting of both KRAS and AKT1 is essentially important to inhibit proliferation of *KRAS* mutated DLD1. Our results showed that miR-4689 effectively reduced phosphorylation of MEK/ERK and dramatically induced proapoptotic factors, including BAX, BAD, BAK, and Cyt C (**Figures 5** and **6**), which almost completely blocked cell proliferation *in vitro* (**Figure 2b**) and *in vivo* (**Figure 6c**). The growth inhibitory effect of miR-4689 was much stronger than that of a siRNA-mediated knock-down of either MEK1 or MEK2; even though the ERK phosphorylation levels were similar. This result suggested that miR-4689 could affect multiple targets simultaneously (**Figure 5b**).

We found that the expression level of miR-4689 was significantly reduced in mutant *KRAS* and mutant *BRAF* cell lines compared with either wild-type cell line although we had only two *KRAS* or *BRAF* wild CRC cells examined. In clinical specimens, we found that miR-4689 was significantly down-regulated in human CRC samples compared with those of normal mucosa samples; moreover, miR-4689 expression was much lower in cancer tissues that harbored mutant *KRAS* compared with those that harbored wild-type *KRAS*. Moreover, mechanistic studies showed that overexpression of *KRAS* gene decreased miR-4689 expression in HEK293 and MRC5 cells (see **Supplementary Figure S2b**), and that knockdown of *KRAS* gene in DLD1 induced miR-4689 expression (see **Supplementary Figure S3a**). These findings suggested that mutant *KRAS/BRAF* CRC cells may have a mechanism to maintain low levels of the potentially toxic miR-4689. Given that the restoration of tumor suppressive miRNAs is a relatively safe, effective strategy for cancer treatment,^40,41^ systemic or focal administration of miR-4689 represents a novel therapeutic strategy for treating mutated *KRAS* cancers. Indeed, we did not detect any serious adverse effects in miR-4689 treated mice (**Figure 7**).

Several siRNA- and miRNA-based therapeutic methodologies have been advanced for delivering these drugs to liver and kidney, and these are undergoing phase 1 or 2 trials for treating hypercholesterolemia, acute kidney injury, liver cancer, transthyretin-mediated amyloidosis, and hepatitis C virus.^42,43^ However, systemic delivery technology of siRNAs and miRNAs for treating solid tumors has been impeded by many limitations.^44^ In this study, we applied a potential *in vivo*, pH sensitive delivery system for miRNAs based on super carbonate apatite nanoparticles.^29,30^ We showed that a systemic administration of miR-4689 mixed with the nanoparticles in nude mice inhibited the growth of pre-established DLD1 (KRAS^G13D^) xenografts compared to a comparable miR-NC administration. After treatment, xenograft samples exhibited a downregulation of KRAS and AKT1 on Western blots. The proapoptosis and antiapoptosis changes observed *in vitro* were also observed *in vivo*.

**Figure 8:**
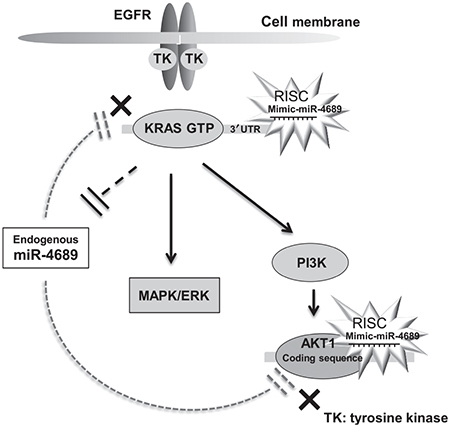
**miR-4689 regulates EGFR signaling pathway.** A simplified scheme illustrating that exogenous miR-4689 can inhibit EGFR signaling through direct inhibition of both KRAS and AKT1. Endogenous miR-4689 expression is normally suppressed in mutated *KRAS* CRC.

In conclusion, we demonstrated that miR-4689 potently suppressed oncogenic *KRAS*-driven EGFR signaling pathways through direct inhibition of KRAS and AKT1 (**Figure 8**). These results may open up new possibilities for a miRNA targeted therapy against intractable mutated *KRAS* CRC.

## Materials and methods

*Cell line.* HEK293, MRC5, and human CRC cell lines, including DLD1, HCT116, SW480, LoVo, RKO, HT29, COLO201, and CaCO-2 were obtained from the American Type Culture Collection (Rockville, MD). CaR-1 with wild-type *KRAS* or *BRAF*^45^ was obtained from JCRB Cell Bank (Japanese Collection of Research Bioresources Cell Bank). All cells were cultured in Dulbecco's modified Eagle's medium, which contained 10% fetal bovine serum, 100 U/ml penicillin, and 100 mg/ml streptomycin. Cultured were incubated at 37°C in a humidified incubator of 95% air and 5% CO_2_.

*Transient miRNA/siRNA/plasmid transfection.* Mature miRNA oligonucleotides (miRs) and miR-NCs were obtained from Gene Design (Osaka, Japan). Small interfering (si) RNA oligonucleotides that specifically targeted MEK1 and MEK2 mRNAs (siMEK1, siMEK2) were obtained from Cell Signaling Technology (Beverly, MA). siRNA oligonucleotides that specifically targeted AKT1 mRNA (siAKT1) and scrambled negative control siRNA were purchased from OriGene Technologies (Rockville, MD). We used pCMV6-Entry plasmid inserted the KRAS^G12V^ and AKT1 sequence with SgfI-MluI restriction sites and pCMV6-Entry plasmid as the control (OriGene, Rockville, MD). Cells were transiently transfected with Lipofectamine RNAiMax or Lipofectamine 2000 (Invitrogen, Carlsbad, CA) according to the protocol provided by the manufacturer.

*shKRAS.* The double-stranded short hairpin RNA clone specifically targeting of *KRAS* gene was purchased from Broad Institute (Cambridge, MA). pLKO.1 vector nontarget short hairpin RNA was used as a control vector. KRAS short hairpin RNA was introduced by lentiviral delivery and selected with puromycin.

*RNA extraction.* Total RNA, including the small RNA fraction, was isolated with the miRNeasy kit (Applied Biosystems, Carlsbad, CA) according to the manufacturer's protocol. Total RNA concentration and purity were assessed with a NanoDrop ND-1000 spectrophotometer (NanoDrop Technologies, Wilmington, DE), at 260 and 280 nm (A_260/280_) wavelengths.

*miRNA microarray analysis.* The samples comprised purified RNAs extracted from HEK293 cells transfected with the Control vector (HEK293-Ctrl) or with the KRAS^G12V^ vector (HEK293-KRAS); or from MRC5 cells transfected with the Control vector (MRC5-Ctrl) or with the KRAS^G12V^ vector (MRC5-KRAS). Extracted total RNA was labeled with Hy5 with the miRCURY LNA Array miR labeling kit (Exiqon, Vedbaek, Denmark). Labeled RNAs were hybridized to 3D-Gene Human miRNA Oligo chips (v.17.0; Toray Industries, Tokyo, Japan). The annotations and oligonucleotide sequences of the probes conformed to the miRBase miRNA database, Release 17 (http://www. microrna.sanger.ac.uk/sequences/). After stringent washes, fluorescent signals were scanned with the 3D-Gene Scanner (Toray Industries) and analyzed with 3D-Gene Extraction software (Toray Industries). The raw data of each spot was normalized to the mean intensity of the background signal, which was determined from blank spot signal intensities and 95% confidence intervals. Measurements of duplicate spots with signal intensities >2 SD above the background signal intensity were considered valid. The relative expression level of a given miRNA was calculated by comparing the signal intensities of the averaged valid spots to their mean value throughout the microarray experiments after normalization by their median values adjusted equivalently. miRNA microarray data has been approved and assigned GEO accession numbers (GEO accession no. GSE60370).

*Real-time, quantitative PCR analysis of messenger RNA expression.* Complementary DNA was synthesized from 1.0 μg total RNA with the High Capacity cDNA Reverse Transcription Kit (Applied Biosystems, Foster City, CA), according to the protocol provided by the manufacturer. qRT-PCR was performed with specifically designed oligonucleotide primers and the LightCycler 480 Real-Time PCR system (Roche Diagnostics, Mannheim, Germany). The amplification products were detected with the LightCycler-DNA master SYBR green I (Roche Diagnostics), according to standard protocols, and the level of target gene expression was calculated. The expression of the target gene was normalized relative to the mRNA expression levels of actin-beta (ACTB), which was used as an endogenous control. The specifically designed PCR primers were, as follows: ACTB forward primer 5′-GATGAGATTGGCATGGCTTT-3′, ACTB reverse primer 5′-CACCTTCACCGTTCCAGTTT3′; KRAS forward primer 5′-ATTCCTTTTATTGAAACATCAGCA-3′, KRAS reverse primer 5′-TCGGATCTCCCTCACCAAT-3′; and AKT1 forward primer 5′-AACACCATGGACAGGGAGAG-3′, AKT1 reverse primer 5′-CATCTTGGTCAGGTGGTGTG-3′.

*Real-time qRT-PCR for miRNA expression.* The reverse transcription reaction was performed with the TaqMan MicroRNA RT Kit (Applied Biosystems, Foster City, CA). Real-time quantitative PCR was performed with TaqMan MicroRNA Assays (Applied Biosystems) and the 7500HT Sequence Detection System (Applied Biosystems). The target miRNA signal was normalized relative to that of the endogenous control, RNU48. Data were analyzed according to the comparative *C*_t_ method.^46^

*Serum response element/AP1 luciferase reporter assay.* All cells were seeded onto 96-well plates and transfected with the SRE luciferase reporter vector (pGL4.33[luc2P/SRE/Hygro]; Promega, Madison, WI) or with the AP1 luciferase reporter vector (pGL4.44[luc2P/AP1 RE/Hygro]; Promega) or with the empty promoter binding site luciferase reporter (pGL4.20[luc2/Puro]; Promega) and Lipofectamine 2000 (Invitrogen) in OptiMEM reduced serum media (GIBCO, Invitrogen, Carlsbad, CA). The transfection efficiency was evaluated with a Renilla luciferase reporter vector (pRL-CMV, Promega). SRE and AP1 transcriptional activities were determined in dual luciferase reporter assays. Twenty-four hours after cotransfection with both reporter vectors and the target miRNA, cells were collected and cell extracts were prepared with the reporter lysis buffer from the Luciferase Assay System (Promega). The luciferase-mediated luminescence was measured with the Dual-Luciferase Reporter Assay System (Promega) according to the manufacturer's instructions. All transfection experiments were conducted in triplicate.

*Proliferation assays.* Cells were seeded at a density of 56 × 10^4^ cells/well in 24-well dishes, and cultured for 24–72 hours to determine proliferation. Cell counting was performed at 24, 48, and 72 hours after transfection or seeding. Counts of cells transfected with miRs were compared to counts of cells transfected with miR-NC.

*Clinical samples.* CRC samples were collected from 46 patients (12 with mutant *KRAS* CRC and 34 with wild-type *KRAS* CRC) that were undergoing surgery at Osaka University Hospital between 2010 and 2013. **Supplementary Table S3** shows the characteristics of these 46 patients. The tumor samples were stored at −80 °C with RNAlater until RNA extraction. All patients gave written informed consent, in accordance with the guidelines approved by the Institutional Research Board of each institute. This study was conducted under the supervision of the Ethics Board of Osaka University Hospital.

*pmirGLO reporter plasmid construction.* The 3′-UTR of human KRAS mRNA was amplified by RT-PCR. The primer sequences were, as follows: Forward—GCTCGCTAGCCTCGATGTCATCTTGCCTCCCTAC; Reverse—ATGCCTGCAGGTCGAATTGGGCAGCAAAGAGATG. The amplified product (254 bp) was subcloned and ligated into the Sal I and Xho I sites in the pmirGLO Dual-Luciferase miRNA Target Expression Vector (Promega) with the In-Fusion HD Cloning Kit (Clontech, Mountain View, CA). The entire sequence (insert and vector) was confirmed by DNA sequencing.

The coding sequence of AKT1 mRNA, including the miR-4689 binding site, was amplified by RT-PCR. The primer sequences were, as follows: Forward—GCTCGCTAGCCTCGAATGAGCGACGTGGCTATTG; Reverse—ATGCCTGCAGGTCGACGCCACAGAGAAGTTGTTG. The amplified product (175 bp) was subcloned and ligated into the *Sal* I and *Xho* I sites in the pmirGLO Dual-Luciferase miRNA Target Expression Vector (Promega) with the In-Fusion HD Cloning Kit (Clontech). The entire sequence (insert and vector) was confirmed by DNA sequencing.

*pmirGLO luciferase reporter assay.* Luciferase assays were conducted with 1 × 10^4^ DLD1 (KRAS^G13D^) cells per well in 96-well plates. Transfections were performed with Lipofectamine 2000 (Invitrogen) in OptiMEM reduced serum media (GIBCO, Invitrogen). Cells were transfected with 100 ng of a pmirGLO construct, which contained either the 3′-UTR of KRAS or the coding sequence of AKT1, and 5 pmol of either miR-NC or miR-4689. Thirty-six hours after transfection, cells were assayed for both firefly and renilla luciferase with the Dual-Luciferase Reporter Assay System (Promega), according to the manufacturer's instructions. All transfection experiments were conducted in triplicate.

*Western blot analysis.* Western blot analysis was performed as described earlier.^47^ Whole cells were lysed in RIPA buffer, containing phosphatase inhibitor and protease inhibitor cocktail. Antibodies against BRAF, MEK1/2, phosphorylated MEK1/2, ERK, phosphorylated ERK, AKT1, phosphorylated AKT, STAT3, BAD, BAX, BAK, BCL2, BCLXL, and Cyt C were obtained from Cell Signaling Technology, Beverly, MA. Antibodies against KRAS and ACTB proteins were obtained from Sigma-Aldrich, St. Louis, MO.

*TUNEL assay.* Apoptotic DNA fragmentation within the cell was analyzed with an *in situ* DeadEnd Fluorometric TUNEL System Assay Kit (Promega), according to the manufacturer's instructions. In brief, 2 × 10^5^ cells/well were plated on Lab-Tek II CC2 Chamber Slides and treated with 50 nM miR-4689 for 48 hours. Cells were fixed in 4% paraformaldehyde at 4°C for 30 minutes, permeabilized in 0.1% Triton X-100, and labeled with fluorescein-12-dUTP with terminal deoxynucleotidyl transferase. The localized green fluorescence of apoptotic cells (fluorescein-12-dUTP) was evaluated by measuring fluorescence intensity with BZ-9000software (KEYENCE, Japan).

*In vivo tumor growth.* DLD1 (KRAS^G13D^) cells were mixed with Matrigel (BD Biosciences, San Jose, CA) and medium at a 1:1 ratio (vol:vol). Cells (2 × 10^6^) in 100 μl of medium/Matrigel solution were injected subcutaneously into both sides of the lower back region of female nude mice (NIHON CLEA, Tokyo, Japan). With carbonate apatite as vehicle, formulated miRNA (40% conjugation)^48^ was administered intravenously (40 μg per injection)^28,29,30^; all injections were delivered via the tail vein, after tumor volumes reached ~75–80 mm^3^. Mice were treated eight times with formulated miR-4689 or miR-NC, three times a week. Mature hsa-miR-4689 sense (5′-UUGAGGAGACAUGGUGGGGGCC-3′) and antisense (5′-GGCCCCCACCAUGUCUCCUCAA-3′), and negative control sense (5′-AUCCGCGCGAUAGUACGUA-3′) and antisense (5′-UACGUACUAUCGCGCGGAU-3′) were obtained from Gene Design (Osaka, Japan). Tumor volumes were determined as described earlier.^30^ All animal experiments were performed in accordance with currently prescribed guidelines and with a protocol approved by Osaka University.

*Statistical analysis.* Each experiment was repeated at least three times. Data are expressed as mean ± SEM. Mean values were compared with the Student's *t*-test. Expression of miRs in wild-type and mutant *KRAS* CRC tissue samples were analyzed with the Wilcoxon rank sum test. Expression levels of miRs in normal colonic mucosa and CRC tissue samples were analyzed with the Wilcoxon signed-rank test. Sequential cell proliferation and tumor growth were analyzed with two-way ANOVAs for repeated measures. Discrete variables were assessed with Fisher's exact test. *P* values ≤0.05 were considered statistically significant. Statistical analyses were performed with JMP software version 9.0 (SAS Institute, Cary, NC).

**SUPPLEMENTARY MATERIAL** **Figure S1.** Comparison of miRNA microarray results between KRAS^G12V^ overexpressed and negative control transfected cells. **Figure S2.** Overexpression of KRAS gene decreased miR-4689 in HEK293 and MRC5. **Figure S3.** The knockdown effects of KRAS to the expression of miR-4689, the ERK phosphorylation, and cell proliferation in KRAS mutated DLD1 cell line. **Figure S4.** Immunoblots for the expression change of apoptosis-related molecules under knockdown of KRAS or AKT1. **Figure S5.** The effects of KRAS- or/and AKT1- overexpression on the inhibition of cell proliferation by miR-4689. **Table S1.** miRNAs down-regulated in HEK293-KRAS compared to HEK293-Ctrl. **Table S2.** miRNAs down-regulated in MRC5-KRAS compared to MRC5-Ctrl. **Table S3.** Relationship between KRAS mutations and clinicopathological factors.
