# Supplementary figures and images for: Concurrent Targeting of KRAS and AKT by MiR-4689 Is a Novel Treatment Against Mutant KRAS Colorectal Cancer
Source: Mol Ther Nucleic Acids. 2015 Mar 10;4(3):e231–. doi: 10.1038/mtna.2015.5 (PMC4354340; doi:10.1038/mtna.2015.5)

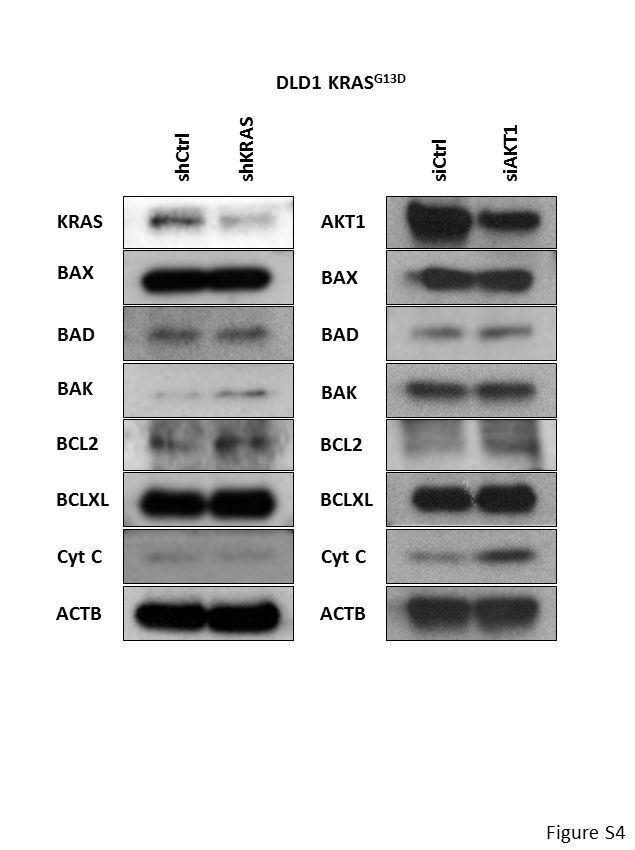

Supplement: Supplementary Information [file mtna20155x1.zip › 2014MTNA000111-s06.tif]

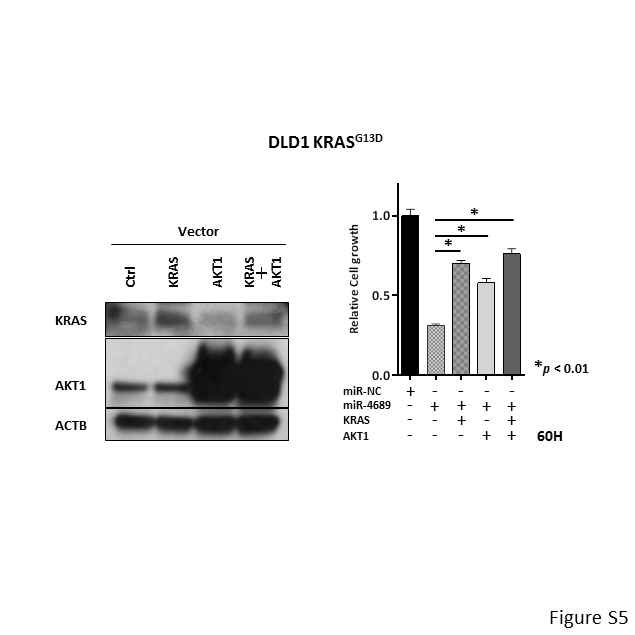

Supplement: Supplementary Information [file mtna20155x1.zip › 2014MTNA000111-s07.tif]

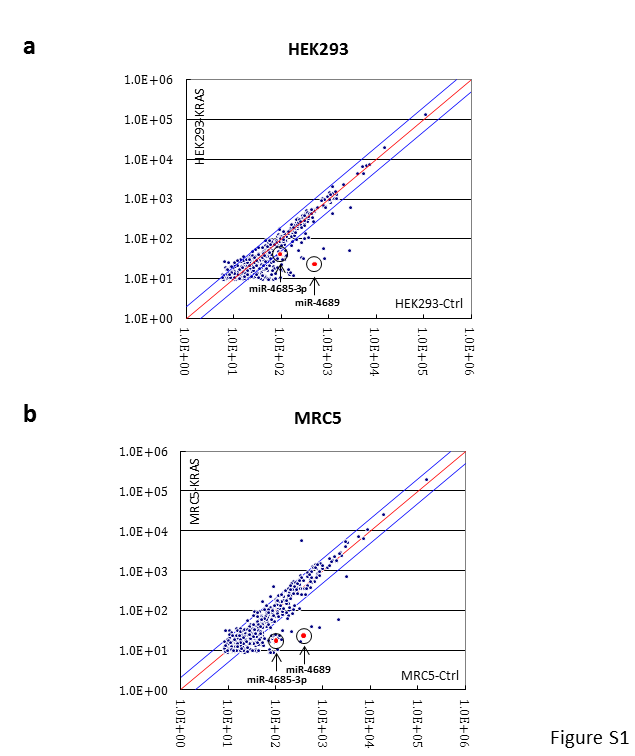

Supplement: Supplementary Information [file mtna20155x1.zip › 2014MTNA000111-s03.tif]

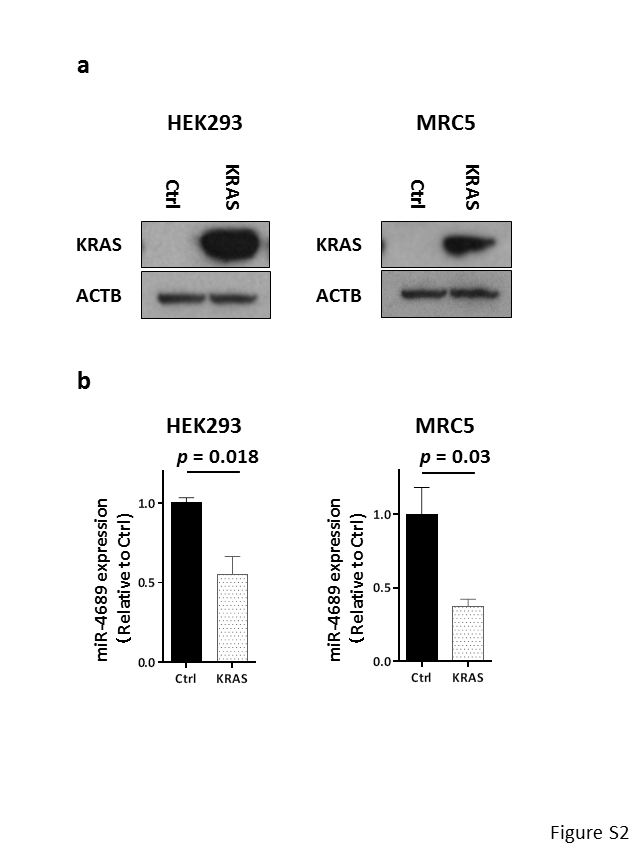

Supplement: Supplementary Information [file mtna20155x1.zip › 2014MTNA000111-s04.tif]

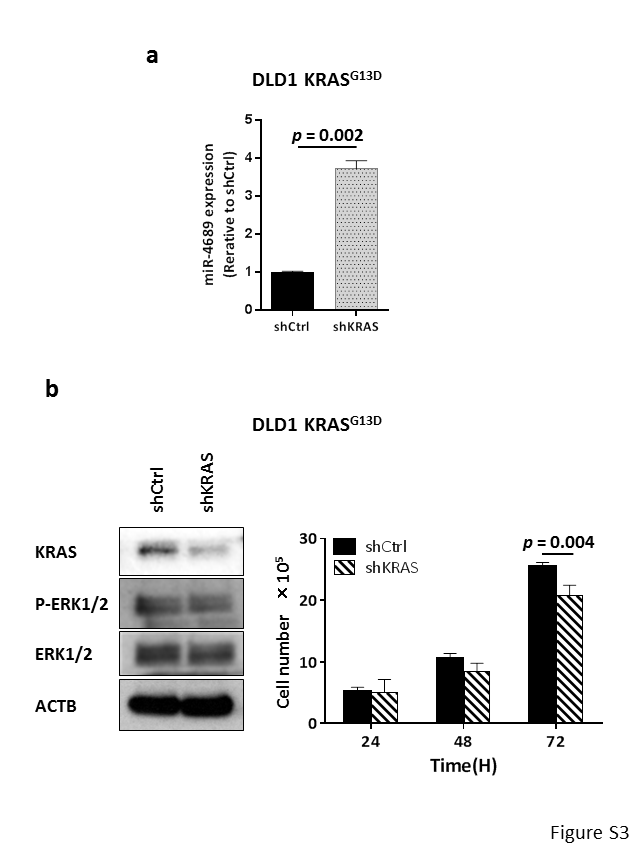

Supplement: Supplementary Information [file mtna20155x1.zip › 2014MTNA000111-s05.tif]
