# Supplementary material for: Concurrent Targeting of KRAS and AKT by MiR-4689 Is a Novel Treatment Against Mutant KRAS Colorectal Cancer
Source: Mol Ther Nucleic Acids. 2015 Mar 10;4(3):e231–. doi: 10.1038/mtna.2015.5 (PMC4354340; doi:10.1038/mtna.2015.5)
Supplement: Supplementary Information [file mtna20155x1.zip › 2014MTNA000111-s08.doc]

**Supplementary Table S1**

**miRNAs down-regulated in HEK293-KRAS compared to HEK293-Ctrl**

| **Rank** | **miR-No.** | **Fold-change  (relative to HEK293‑Ctrl)** | **Ref Seq ID** |
| --- | --- | --- | --- |
| **1** | **hsa-miR-4442** | **53.2** | **MIMAT0018960** |
| **2** | **hsa-miR-4270** | **25.3** | **MIMAT0016900** |
| **3** | **hsa-miR-4689** | **21.5** | **MIMAT0019778** |
| **4** | **hsa-miR-3131** | **15.5** | **MIMAT0014996** |
| **5** | **hsa-miR-296-5p** | **13.7** | **MIMAT0000690** |
| **6** | **hsa-miR-3675-3p** | **11.2** | **MIMAT0018099** |
| **7** | **hsa-miR-2355-5p** | **10.9** | **MIMAT0016895** |
| **8** | **hsa-miR-4731-3p** | **10** | **MIMAT0019854** |
| **9** | **hsa-miR-3619-3p** | **9** | **MIMAT0019219** |
| **10** | **hsa-miR-4274** | **8.9** | **MIMAT0016906** |
| **11** | **hsa-miR-1249** | **8.4** | **MIMAT0005901** |
| **12** | **hsa-miR-3679-3p** | **8.1** | **MIMAT0018105** |
| **59** | **hsa-miR-4685-3p** | **1.33** | **MIMAT0019772** |

**Abbreviations: HEK293-KRAS = HEK293 cells transfected with KRAS^G12V^ Vector; HEK293-Ctrl = HEK293 cells transfected with Control Vector; miR and miRNA = microRNA**
