# Supplementary material for: Concurrent Targeting of KRAS and AKT by MiR-4689 Is a Novel Treatment Against Mutant KRAS Colorectal Cancer
Source: Mol Ther Nucleic Acids. 2015 Mar 10;4(3):e231–. doi: 10.1038/mtna.2015.5 (PMC4354340; doi:10.1038/mtna.2015.5)
Supplement: Supplementary Information [file mtna20155x1.zip › 2014MTNA000111-s09.doc]

**Supplementary Table S2**

**miRNAs down-regulated in MRC5-KRAS compared to MRC5-Ctrl**

| **Rank** | **miR-No.** | **Fold-change  (relative to MRC5-Ctrl)** | **Ref Seq ID** |
| --- | --- | --- | --- |
| **1** | **hsa-miR-4442** | **36.7** | **MIMAT0018960** |
| **2** | **hsa-miR-4270** | **23.2** | **MIMAT0016900** |
| **3** | **hsa-miR-3619-3p** | **20.6** | **MIMAT0019219** |
| **4** | **hsa-miR-4689** | **16.6** | **MIMAT0019778** |
| **5** | **hsa-miR-296-5p** | **14.7** | **MIMAT0000690** |
| **6** | **hsa-miR-4731-3p** | **10.4** | **MIMAT0019854** |
| **7** | **hsa-miR-625*** | **10** | **MIMAT0004808** |
| **8** | **hsa-miR-3614-5p** | **9.1** | **MIMAT0017992** |
| **9** | **hsa-miR-4279** | **8.6** | **MIMAT0016909** |
| **14** | **hsa-miR-4685-3p** | **2.53** | **MIMAT0019772** |

**Abbreviations: MRC5-KRAS = MRC5 cells transfected with KRAS^G12V^; MRC5-Ctrl = MRC5 cells transfected with Control Vector; miR and miRNA = microRNA**
