# Supplementary material for: Concurrent Targeting of KRAS and AKT by MiR-4689 Is a Novel Treatment Against Mutant KRAS Colorectal Cancer
Source: Mol Ther Nucleic Acids. 2015 Mar 10;4(3):e231–. doi: 10.1038/mtna.2015.5 (PMC4354340; doi:10.1038/mtna.2015.5)
Supplement: Supplementary Information [file mtna20155x1.zip › 2014MTNA000111-s10.doc]

**Supplementary Table S3**

**Relationship between KRAS mutations and clinicopathological factors**

| **Clinicopathological factors** | **Wild type KRAS (n=34)** | **Mutated KRAS (n=12)** | ***p-*value** |
| --- | --- | --- | --- |
| **KRAS** |  |  |  |
| **G12C/G12D/G12V/G13D** | **-** | **2/4/4/2** | **-** |
| **Gender** |  |  |  |
| **Male/Female** | **19/15** | **8/4** | **NS** |
| **Tumor size (cm) (mean ± SD)** | **5.5 ± 2.5** | **4.7 ± 1.0** | **NS** |
| **Histological grade** |  |  |  |
| **Well** **or** **Mod/Others** | **29/5** | **12/0** | **NS** |
| **Serosal invasion** |  |  |  |
| **Present/Absent** | **3/31** | **1/11** | **NS** |
| **UICC Stage** |  |  |  |
| **I/II/III/IV** | **2/6/15/11** | **2/1/5/4** | **NS** |
| **Lymphatic invasion** |  |  |  |
| **Present/Absent** | **32/2** | **10/2** | **NS** |
| **Venous invasion** |  |  |  |
| **Present/Absent** | **17/17** | **5/7** | **NS** |
| **Lymph node metastasis** |  |  |  |
| **Present/Absent** | **25/9** | **9/3** | **NS** |

**Abbreviations: well = well differentiated; mod = moderately differentiated; NS = not significant**
