# Supplementary material for: Concurrent Targeting of KRAS and AKT by MiR-4689 Is a Novel Treatment Against Mutant KRAS Colorectal Cancer
Source: Mol Ther Nucleic Acids. 2015 Mar 10;4(3):e231–. doi: 10.1038/mtna.2015.5 (PMC4354340; doi:10.1038/mtna.2015.5)
Supplement: Supplementary Information [file mtna20155x1.zip › 2014MTNA000111-s02.doc]

**Supplementary Figure Legends**

**Supplementary Figure S1.** Comparison of miRNA microarray results between KRAS^G12V^ overexpressed and negative control transfected cells.

A scatter plot of the microarray intensities of KRAS^G12V^ overexpressed cells were plotted against those of negative control transfected cells (**a**) HEK293, (**b**) MRC5.

Each point represents the average of duplicate wells.

**Supplementary Figure S2.** Overexpression of KRAS gene decreased miR-4689 in HEK293 and MRC5. (**a**) Immunoblots showed huge KRAS expression in KRAS transfected HEK293 and MRC5. Ctrl: Control vector transfected cells. (**b**) qRT-PCR assays showed that miR-4689 expression was reduced in KRAS transfected HEK293 and MRC5. Data represent mean ± SEM.

**Supplementary Figure S3.** The knockdown effects of KRAS to the expression of miR-4689, the ERK phosphorylation, and cell proliferation in KRAS mutated DLD1 cell line. (**a**) The expression of miR-4689 was up-regulated in shKRAS mediated DLD1 compared to that in shCtrl (*p* = 0.002). (**b**) (*left*) Immunoblots for KRAS, P-ERK1/2, and ERK1/2 expression in DLD1 cells transfected with shCtrl or shKRAS vector. ACTB is a loading control. (*right*) A significant but only modest growth inhibitory effects was noted in shKRAS-DLD1 compared to shCtrl-DLD1 (*p* = 0.004). All data represent mean ± SEM.

**Supplementary Figure S4.** Immunoblots for the expression change of apoptosis-related molecules under knockdown of KRAS or AKT1. ACTB is a loading control. The whole lysates were used in this experiment.

**Supplementary Figure S5.** The effects of KRAS- or/and AKT1- overexpression on the inhibition of cell proliferation by miR-4689. (*left*) Immunoblots for KRAS and AKT1 expression in KRAS- or/and AKT1- overexpressed DLD1. ACTB is a loading control. (*right*) KRAS- or/and AKT1- overexpression restored miR-4689 mediated growth inhibition in DLD1. Data represent mean ± SEM.
